# Supplementary material for: Cerebrovascular regulation in patients with active tumors and an acute ischemic stroke: a retrospective analysis
Source: Front Physiol. 2024 Dec 19;15:1423195. doi: 10.3389/fphys.2024.1423195 (PMC11693647; doi:10.3389/fphys.2024.1423195)
Supplement: Supplementary file 1 [file Table1.docx]

Cerebrovascular regulation in patients with active tumors and an acute ischemic stroke: A retrospective Analysis

^1^Lehel-Barna Lakatos, MD, ^1^Manuel Bolognese, MD, ^1^Mareike Oesterreich, FND, ^1^Martin Müller, MD, ^2^Grzegorz Marek Karwacki, MD

^1^Department of Neurology and Neurorehabilitation, ^2^Department of Radiology and Nuclear Medicine, Section Diagnostic and Invasive Neuroradiology, Lucerne Cantonal Hospital, 6000 Lucerne, Switzerland

Words 5900 (overall)

Tables 4, Figure 2

Running title: Stroke in patients with active tumors

Key words: stroke, malignancy, hypertension, cerebral autoregulation, stroke volume, magnetic resonance imaging, Doppler ultrasound.

Correspondence

Prof Dr Martin Müller

Department of Neurology and Neurorehabilitation

Lucerne Cantonal Hospital

Spitalstrasse

CH-6000 Lucerne

Email: [martin.renatemueller@web.de](mailto:martin.renatemueller@web.de)

Abstract

Ischemic stroke in patients with a systemic tumor disease or cancer not in remission (active tumors) is less well understood. Some aspects of such paraneoplastic strokes remind on a generalized cerebrovascular disorder. We hypothesized that cerebrovascular regulation in active tumor patients with a stroke is different from other patients with stroke who have no active tumor disease. Within the first 72 hours after the acute ischemic stroke, cerebral blood flow regulation was analyzed by means of transfer function analysis between middle cerebral artery blood flow velocity and blood pressure with estimation of coherence, gain and phase in the very low (0.02-0.07 Hz), low (0.07-0.20 Hz) and high frequencies (0.20-0.5 Hz) in four stroke groups: active tumors, inactive tumors (untreated and in remission), hypertensive lacunar stroke (LS), and non-hypertensive embolic stroke (NHES). The 4 groups did not differ regarding age, sex distribution, and brain infarct size on magnet resonance imaging Between the four stroke groups, phase was not different in any frequency range in both hemispheres. Gain was highest (either significant or by trend) in the active tumor group in the HF range in comparison to all other stroke subgroups, it was also higher in the LF range in the stroke affected hemisphere when compared to the LS group. The HF gain findings were independent of end-tidal CO2 levels but exhibited some dependency of coherence. The high gain can be interpreted as a generalized high vascular resistance. The cerebrovascular regulation in active tumor patients seems to exhibit some analogy to hypertensive patients with lacunar stroke.

ClinicalTrials.gov NCT04611672, 11.10.2020

**Introduction**

Ischemic stroke can grossly be classified into cerebral ischemia in the distribution of a cortical artery (territorial infarction; figure 1A) or in the vicinity of a small subcortical artery not being larger than 1.5 cm in diameter (lacunar ischemia, figure 1B; Fazekas et al, 2002). While lacunar strokes are associated nearly exclusively with arterial hypertension and other vascular risk factors, the etiology of territorial strokes is wide and includes cardiac embolism due to different cardiac diseases, large artery disease due to atherosclerotic lesions, some vasculitis disorders, or coagulopathies [Adams et al, 1993]. Very often a distinct cause of a territorial stroke is not identified. Patients with tumors or cancer can be divided into those in remission (inactive tumor patients) and into those with clinical signs of tumor/cancer activity including being under treatment (active tumor patients). Specifically in active tumor patients with systemic extracranial tumors and no accompanying intracranial tumor manifestation, the pathophysiology of stroke is - in the absence of more common causes of stroke (cardiac embolism, large vessel disease and cerebral microangiopathy) - not well understood [Dardiotis et al, 2019]. Many strokes in these patients show imaging findings in agreement with embolic stroke [Bang et al, 2020; Navi et al, 2021] and an involvement of multiple vessels in different territories of the brain supplying arteries (figure 1C). The involvement of multiple brain arteries is suggestive of a process affecting the brain arteries in a generalized manner, for example via tumor associated systemic inflammatory processes or coagulation disturbances [Dardiotis et al, 2019; Bang et al, 2020; Navi et al 201]. Clinically, patients with active systemic tumors exhibit a poorer long term neurological prognosis and a higher risk of stroke recurrence [Navi et al, 2021]. Such a clinical course provides some analogies to the well-known course of cerebral microangiopathy due to arterial hypertension with its clinical deterioration over time and recurrent stroke events [Markus and de Leeuw, 2023; Hainsworth et al, 2023].

One could consider that such clinical differences could be caused by cerebral blood flow (CBF) regulation disturbances. The investigation of CBF regulation with the transfer function analysis (TFA) method [Panerei et al, 2022] provides information on the dynamics of cerebral autoregulation (dCA) over a frequency range of 0.02 Hz to 0.5 Hz, corresponding to CBF changes occurring between 50 and 2 seconds. The target variables of TFA are coherence, gain and phase. Coherence describes the stability over time of the relation between arterial blood pressure (BP) and CBF velocity (CBFV, as assessed by transcranial Doppler sonography), gain the transmural power transmission between both, and phase the phase shift between the two. Compared to healthy subjects, the main finding in non-tumor-associated strokes is that the phase between CBF(V) and BP is reduced in the frequency range of 0.02 – 0.07 Hz (very low frequency, VLF) or of 0.07-0.15 Hz (low frequency, LF) while gain and coherence have been less affected [Nogueira et al, 2022]. In the high frequency range (0.15-0.5 Hz) coherence, gain and phase are usually not relevantly changed. Arterial hypertension affects the brain arteries in a generalized manner. If assessed by the dCA assessing techniques [Panerai et al, 1999; Ainslie et al, 2008; Müller et al, 2014]; Panerai et al, 2022; Nogueira et al, 2022] and compared to healthy controls, patients with untreated hypertension [Lipsitz et al, 2000; Serrador et al, 2005; Zhang et al, 2007] and treated patients with a long history of hypertension [Müller et al, 2019] show distinct changes in the regulation of cerebral blood flow best explained by differences in the vascular tone. We investigated in this retrospective study on patients with extracranial tumor disease (without intracranial tumor manifestation) and an acute ischemic stroke how their cerebral blood flow (or its velocity, CBFV) is regulated in terms of dCA. We hypothesized, that CBFV regulation in terms of phase, gain and coherence in active tumor patients with a stroke are different from other patients with stroke who have no active tumor disease. We compared acute stroke patients with extracranial tumor disease to a) patients with a pure lacunar stroke due to hypertension (lacunar stroke, LS) as a model of a known generalized brain artery disorder [Pires et al, 2013], and b) to non-hypertensive patients with embolic strokes (NHES) as a counter model without hypertensive microangiopathy.

Figure 1. Examples of the characteristic diffusion weighted imaging (DWI) pattern of the three stroke groups investigated in this study. In DWI an acute ischemic stroke is indicated by bright/white color on the image (arrows) in contrast to the intact dark brain tissue. 1A, embolic cortical territorial infarction; 1B, hypertensive lacunar infarction; 1C, multiple small left hemispheric cortical infarction of a patient with a pancreatic carcinoma.


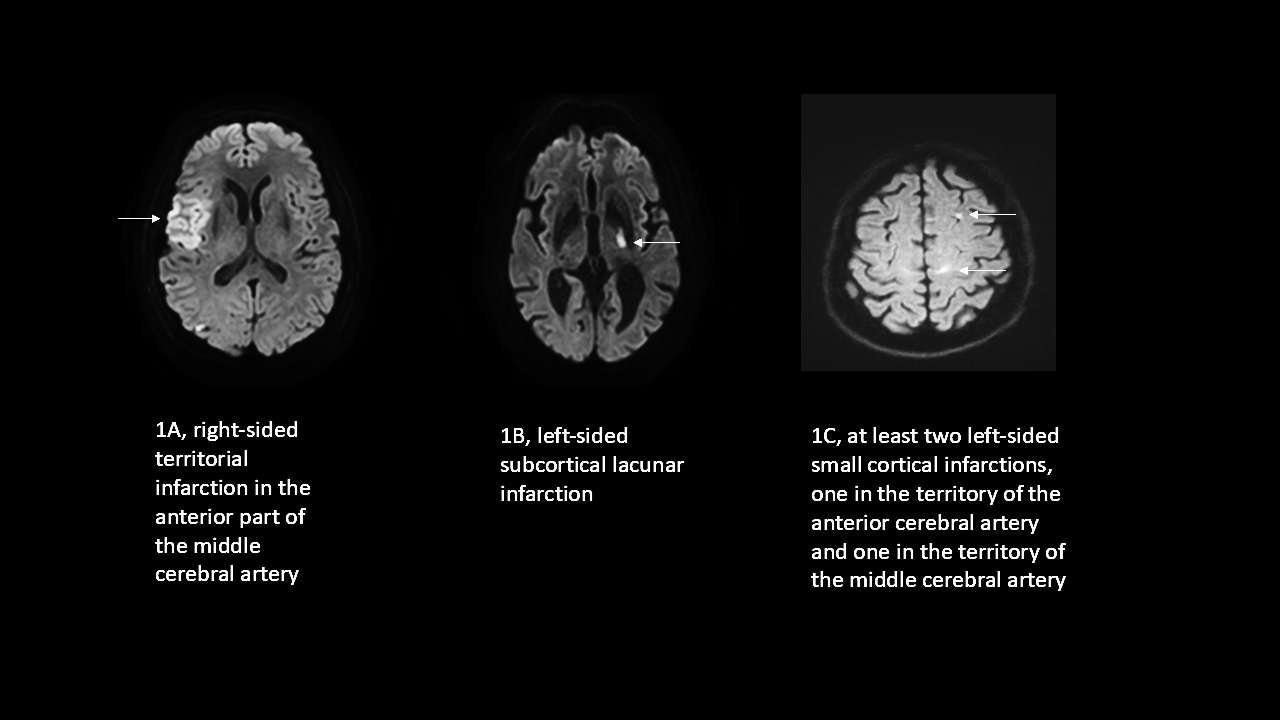


**Material and Methods**

The study was approved by the Ethics Committee of Northwest and Central Switzerland, was conducted adhering to the Declaration of Helsinki, using good standards of clinical practice, and is part of a larger trial (registered at ClinicalTrials.gov NCT04611672) in which routine data from stroke unite patients were collected prospectively between January 1, 2020, until April 31, 2022 . The corresponding author can provide all the data upon reasonable request.

The Lucerne Hospital is a large tertiary teaching hospital with a complete stroke center service. All patients with a stroke syndrome receive standardized care, with initially a focused clinical examination followed by a multimodal cranial computed tomography [native CT; perfusion CT, postprocessed by Syngo.via (Siemens, Germany) and Rapid CTP (RAPID AI, USA)], and CT angiography. If indicated, intravenous thrombolysis and/or arterial thrombectomy follow immediately. All patients diagnosed with stroke syndrome are transferred to the stroke unit for intensive clinical monitoring. The monitoring includes National Institute of Health Stroke Scale (NIHSS) [Lyden et al, 1994] and modified Rankin score (mRs) [van Swieten et al, 1988] assessments upon hospital entry, as well as daily assessments while on the stroke unit and three months after the ischemic event. Blood pressure, heart rate, body temperature, blood glucose levels, and oxygen saturation are closely monitored. Extensive ultrasound examinations of all brain-supplying arteries (including dCA assessment), an echocardiogram, and brain magnetic resonance imaging (MRI) with DWI, T2, and SWI sequences on either a Vida fit (3 Tesla, Siemens, Germany) or aa Aera (1,5 Tesla, Siemens, Germany) or an Achieva (3 Tesla, Philips, Netherlands) follow within 72 hours after hospitalization. Infarct size estimation on MRI was calculated by the ABC/2 method, which demonstrated in our hands a good agreement with automatic software [Lakatos et al, 2022]. For this work, we additionally classified the cortical infarcts into small if the largest lesion diameter was 2 cm or less, and into large if the largest lesion diameter was greater than 2 cm (the 2 cm limit was arbitrarily chosen). Lacunes were defined as infarcts with a diameter of 1.5 cm or less present in the subcortical structures (basal ganglia, white matter) [Fazekas et al 2002]. Patients whose neurological deficit resolved within 24 hours and whose DWI remained negative were classified as having suffered from a TIA.

Patients

For this study, we retrospectively analyzed all patients treated at our stroke unit from January 1, 2020, to April 31, 2022. Inclusion criteria were: age over 18 years, absence of pregnancy, the presence of a characteristic hemispheric syndrome diagnosed as a definitive supratentorial ischemic stroke in the middle cerebral artery territory after initial multimodal imaging and later confirmed by DWI imaging, the presence of an ischemic stroke in only one hemisphere to classify the results into belonging to the affected hemisphere (AH) and the unaffected hemisphere (UH), no secondary severe bleeding into the infarcted area to avoid recordings under the influence of hemorrhage caused space occupying lesions, TCD recordings of good quality in the middle cerebral artery at a depth of 45-60 mm, at least of the AH, and determination of dCA within 48 hours of the stroke event. Exclusion criteria included the final diagnosis of a stroke mimic, a primary intracranial hemorrhage, a TIA, as well as a cerebral sinus or vein thrombosis, or the presence of a brain metastasis or a primary brain tumor as a cause of the neurological deficit.

dCA assessment

We performed all investigations with the subject in a resting supine position with the head elevated by approximately 30°. The spontaneous fluctuations of middle cerebral artery CBFV (2 MHz probe; MultidopX, DWL; Compumedics, Sipplingen, Germany) and BP (Finometer Pro; Finapres Medical Systems, Amsterdam, The Netherlands) were simultaneously recorded for at least 6 minutes. End-tidal pCO2 (EtCO2) concentration was measured via nostril tubes and the capnograph embedded in the TCD device. BP, CBFV, and EtCO2 data were collected at 100 Hz. The data were analyzed using Matlab (2023a; Math Works Inc., Natick, MA, USA). The data were visually inspected for artifacts, and only artifact-free data periods of 5 minutes were used for analysis. Each raw data time series was averaged over a 1-second interval to create a new time series with fewer data points. The coherence, phase and gain between the new BP and CBFV time series were extracted from their respective power auto-spectra or cross-spectra using Welch’s averaged periodogram method, with a Hanning window length of 100 s, window overlap of 50%, and total Fast Fourier transformation data length of 300 seconds. For each subject, the coherence, the phase (in radians), and the gain (in cm/s/mmHg) were averaged over the VLF, LF and HF ranges. Severe wrap-arounds in the frequency range <0.1 Hz were removed before averaging. For averaging we used only those values whose coherence was ≥ 0.4.

Statistics

For all data analysis, the Matlab Statistical Toolbox was used. Normally distributed data are reported as mean ± SD, not normally distributed data as median with their interquartile range (IQR). Most continuous variables were not normally distributed. We considered all patients as one stroke population with four subgroups, and used nonparametric One-Way ANOVA analog Kruskal-Wallis-test for all between group comparisons for continuous variables. Fisher’s exact test, or chi^2^ statistics, was used to compare categorical variables. Regression analysis was used to evaluate whether EtCO2 or coherence confounded the dCA parameter gain and phase within each stroke group (the reported r^2^ values are the adjusted r^2^ values). Finally, fixed effect ANCOVA models were used to test whether the covariate (either ETCO2 or coherence) affected phase or gain differently between the four stroke groups. P <.05 was considered statistically significant.

**Results**

Patients

During the reported period, we treated a total of 337 patients who fulfilled the inclusion criteria, particularly the dCA assessment of good quality. Among them, 39 patients had a history of an extracranial tumor disease (Figure 2), with 20 suffering from active tumors, and 19 having inactive tumorsafter adequate treatment. We compared these patients with two kinds of stroke cohorts: one consisting of 45 patients with a pure LS due to hypertension as their most likely stroke cause; the second cohort consisting of 39 NHES patients who had had a similar age and sex distribution, and not significant different MRI infarct volumes. Clinically, the four stroke cohorts were comparably balanced regarding the cerebrovascular risk factors (apart from arterial hypertension). As to be expected large artery disease, intravenous and mechanical thrombolysis were less present in the hypertensive pure LS group. (Tables 1 and 2). Within the tumor patients, stroke severity and 3 months outcome were not different. Notably, the NHES group exhibited significantly more left hemispheric infarctions than the tumor patients and the LS patients.

In patients with active tumors coagulopathies are suggested to be causative for the strokes [Navi et al, 2021]. As gross markers of the state of coagulation, we found the International Normalized Ratio (INR) and the Partial Thromboplastin Time (PTT) not different between the four stroke groups [LS group, INR 1 (IQR 1 - 1.1), PTT 30 (IQR 27 – 32); NHES group, INR 1 (0.9 – 1.1), PTT 30 (28 – 34); inactive tumors, 1.1 (0.9 – 1.1); active tumors, INR 1.1 (1.0 – 1.2), PTT 30 (27 – 33). P-value for INR 0.68, for PTT 0.14]

Figure 2: Patients recruitment


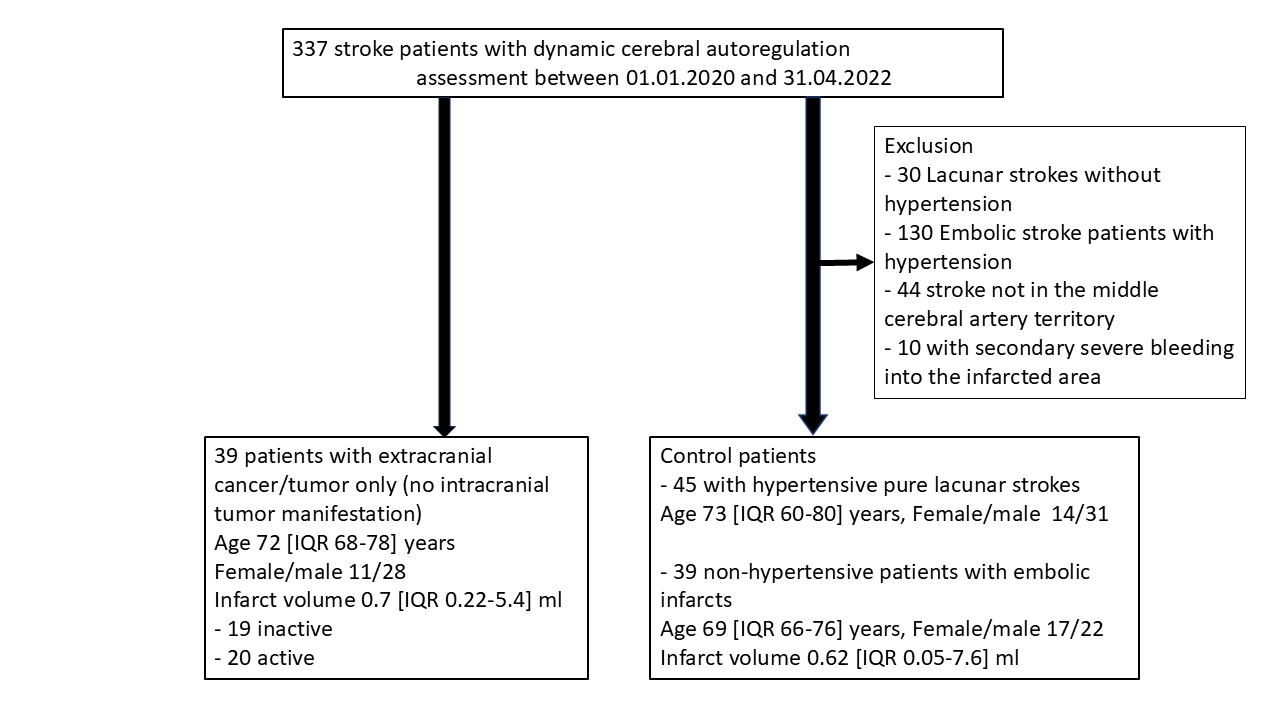


Table 1: Basic clinical characteristics of the three different stroke cohorts.

| Variable | Active tumor patients  N=20 | Inactive tumor patients N=19 | Hypertensive patients with pure lacunar stroke (LS)  N=45 | Non-hypertensive patients with embolic infarcts (NHES)  N=39 | P-value |
| --- | --- | --- | --- | --- | --- |
| Female/male | 7/13 | 4/15 | 14/31 | 17/22 | p=.36 |
| Age in years | 71 [67 - 76] | 75 [64 - 79] | 73 [60 - 80] | 69 [66 - 76] | p=.23 |
| Tumors | Bronchial Ca 7  Mamma Ca 2  Polycytemia vera JAK2 positive 2  Pancreatic Ca 2  Oropharyngeal Ca 1  Non hodgkin lymphoma 1  Hepatocellular 1  Esophageal 1  Heart sarcoma 1  Retroperitoneal sarcoma 1  Chronic myeloid leukemia 1 | Prostatic Ca 4  Mamma Ca 3  Urothelial Ca 3  Chronic Lymphoma 2  Malignant Melanoma 2  Renal cell Ca 1  Bronchial Ca 1  Basal cell Ca 1  Pharyngeal Ca 1  Neuroendocrine Tumor 1 | 0 | 0 |  |
| Arterial Hypertension | 16 | 14 | 45 (by definition) | 0 (by definition) | p=.0000 |
| Diabetes mellitus | 3 | 2 | 11 | 12 | p=.27 |
| Dyslipidemia | 16 | 15 | 40 | 31 | p=.45 |
| Body mass index | 24.2 [21.1 – 25.7] | 25.9 [23.7 - 28.5] | 26.4 [23.4 – 19.4] | 24.4 [22.8 – 28.7] | p=.05  Subgroup analysis: NHES vs inactive tumor p=.04 |
| Active Smoking | 2 | 8 | 15 | 8 | p=.18 |
| Atrial fibrillation | 6 | 3 | 5 | 3 | p=.17 |
| Large vessel disease | 3 | 4 | 0 | 6 | p=.15 |
| Coronary artery disease | 2 | 6 | 7 | 6 | p=.89 |
| Stroke infarct volume (ml) on MRI | 1.03 [0.40 -5.0] | 0.42 [0 – 7.75] | 0.30 [0.06 - 0.65] | 0.62 [0.05 - 7.6] | p=.34 |
| Stroke affected hemisphere right/left | 10/10 | 9/10 | 21/24 | 9/30 | p=.02 |
| Iv lysis | 4 | 4 | 6 | 14 | p=.04 |
| Mechanical thrombectomy | 2 | 3 | 0 | 4 | p=.10 |
| NIHSS on admission | 2.5 [1 - 7] | 4 [0.25 - 7] | 3 [1 - 3.25] | 3 [1 - 5] | p=.85 |
| mRs after 3 months | 1 [0 - 3] | 0 [0 - 2] | 1 [0 - 1] | 1 [0 - 1.25] | p=.95 |

Ca, carcinoma; MRI, magnetic resonance imaging; iv lysis, intravenous thrombolysis; NIHSS, National Institue of Health Stroke Scale; mRs, modified Rankin scale. Data in numbers or in median and interquartile range.

Table 2. Distribution of infarct type among the different patient groups

|  | Hypertensive pure lacunar stroke (N=45) | Non-hypertensive embolic stroke (N=39) | IInactive tumor (N=19) | Active tumor (N=20) |
| --- | --- | --- | --- | --- |
| Lacunar infarctions | 45 | 4 | 2 | 2 |
| Single small cortical infarctions | 0 | 11 | 8 | 2 |
| Multiple small cortical infarctions | 0 | 10 | 4 | 11 |
| Large cortical infarction | 0 | 14 | 5 | 5 |

Cortical infarctions were classified as small if their largest diameter was 2 cm or less; they were classified as large when their diameter was more than 2 cm. Lacunes are small infarcts in the subcortical white matter or in the basal ganglia.

Dynamic cerebral autoregulation

EtCO2, BP and CBFV over the recording period did not differ significantly between the four patient groups. Only for EtCO2 there was a trend to be higher in the LS group compared to the active tumor group (Table 3).

Affected hemisphere

Coherence, was not significantly different between the four groups in all three frequency ranges. Between the four stroke groups, gain was not different in the VLF range. It showed a strong trend in the LF range with the active tumor patients exhibiting a higher gain than the LS patients. HF gain was significantly higher in the active tumor patients compared to the LS, NHES, and the inactive tumor patients. Phase was not significant different between the four stoke groups in all frequency ranges.

Unaffected hemisphere

Coherence was not different in the VLF range between the four groups. LF coherence was significantly lower in the inactive etumor patients compared to the NHES and active tumor patients. HF coherence was lowest in the inactive tumor patients achieving a significant difference to the active tumor patients. Gain was not different in the VLF and LF ranges. HF gain showed strong trends to be highest in the active tumor patients compared to the other three stroke groups. Phase was not different between the 4 stroke groups in neither frequency range

Table 3. Coherence, gain and phase in the stroke affected hemisphere in the different patient groups.

|  | Hypertensive pure lacunar stroke (LS) | Non-hypertensive embolic stroke (NHES) | IInactive tumor | Active tumor | P-value |
| --- | --- | --- | --- | --- | --- |
| Mean arterial blood pressure (mm Hg) | 97 [85 - 107] | 99 [90 - 105] | 95 [(87 - 106] | 92 [84; 99] | Overall p=.38 |
| End-tidal pCO2 (mmHg) | 38.9 [37.5 - 40.5] | 39.4 [38.6 - 41] | 39.4 [38.2 - 40.7] | 40 [39.4; 41.8] | Overall p=.09  Subgroup analysis LS vs active tumor p=.06 |
| CBFV (cm/s) | 43 [38 - 50] | 47 [39 - 58] | 42 [36 - 51] | 48 [37 - 59] | Overall p=.29 |
| Coherence |  |  |  |  |  |
| -VLF | 0.57 [0.48 - 0.66] | 0.57 [0.52 - 0.64] | 0.58 [0.46 0.68] | 0.57 [0.47 - 0.65] | Overall p=.99 |
| -LF | 0.59 [0.50 - 0.73] | 0.63 [0.565 - 0.79] | 0.63 [0.44 - 0.75] | 0.72 [0.65 - 0.78] | Overall p=.12 |
| -HF | 0.70 [0.58 - 0.76] | 0.69 [0.60 -0.77] | 0.66 [0.53 - 0.75] | 0.78 [0.71 - 0.83] | Overall p=.10 |
| Gain (cm/s/mmHg) |  |  |  |  |  |
| -VLF | 0.23 [0.14 - 0.38] | 0.18 [0.13 - 0.40] | 0.17 [0.11 - 0.29] | 0.26 [0.23 - 0.36] | Overall p=.21 |
| -LF | 0.35 [0.22 -0.51] | 0.38 [0.26 - 0.57] | 0.35 [0.22 - 0.42] | 0.47 [0.32 - 0.58] | Overall p=.07  Subgroup analysis:  Active tumors vs LS strokes p=.08 |
| -HF | 0.47 [0.36 -0.59] | 0.46 [0.35 - 0.58] | 0.39 [0.31 - 0.63] | 0.61 [0.48 - 0.72] | Overall p=.03  Subgroup analysis: active tumors vs NHES p=.03  active tumors vs LS .09  active tumor vs inactive tumors p=.04 |
| Phase (radian) |  |  |  |  |  |
| -VLF | 0.84 [0.62 - 1.12] | 0.81 [0.60 - 1.00] | 0.72 [0.51 - 1.06] | 0.70 [0.37 - 1.12] | Overall p=.56 |
| LF | 0.67 [0.49 - 1.01] | 0.68 [0.50 0.96] | 0.68 [0.51 - 0.83] | 0.58 [0.39 - 0.77] | Overall p=.23 |
| -HF | 0.20 [0.05 - 0.38] | 0.24 [0.04 - 0.46] | 0.25 [0.17 - 0.41] | 0.13 [0.04 - 0.25]) | Overall p=.50 |

Overall, Kruskal-Wallis test was used for comparisons over all four groups P -value, level of significance. pCO2, partial pressure of carbon dioxide; CBFV, cerebral blood flow velocity in the middle cerebral artery; VLF, very low frequency; LF, low frequency; HF, high frequency. All data in median and interquartile range.

Table 4. Coherence, gain and phase in the stroke unaffected hemisphere in the different patient groups.

|  | Hypertensive pure lacunar stroke (LS) | Non-hypertensive embolic stroke (NHES) | InInactive tumor | Active tumor | P-value |
| --- | --- | --- | --- | --- | --- |
| CBFV (cm/s) | 44 [38 - 51] | 46 [39 -57] | 43 [37 - 51] | 48 [38 - 58] | Overall p=.31 |
| Coherence |  |  |  |  |  |
| -VLF | 0.56 [0.44 - 0.65] | 0.55 [0.51 - 0.64] | 0.61 [0.30 - 0.66] | 0.54 [0.49 - 0.63] | Overall p=.85 |
| -LF | 0.67 [0.52 - 0.77] | 0.68 [0.54 - 0.80] | 0.55 [0.43 - 0.61] | 0.67 [0.59 - 0.79] | Overall p=.02  Subgroup analysis: NHES vs inactive tumor p=.04  active tumor vs inactive tumor p=.03 |
| -HF | 0.70 [0.55 - 0.79] | 0.68 [0.60 - 0.78] | 0.62 [0.55 - 0.69] | 0.77; [0.67 - 0.82] | Overall p=.08  Subgroup analysis: active tumor vs inactive tumor p=.04 |
| Gain (cm/s/mmHg) |  |  |  |  |  |
| -VLF | 0.24 [0.14 - 0.34] | 0.22 [0.13 - 0.33] | 0.20 [0.13 - 0.30] | 0.30 [0.15 - 0.40] | Overall p=.59 |
| -LF | 0.38 [0.27 - 0.53] | 0.39 [0.29 - 0.52] | 0.39 [0.35 - 0.55] | 0.54 [0.26 - 0.72] | Overall p=.43 |
| -HF | 0.47 [0.38 - 0.61] | 0.48 [0.40 - 0.55] | 0.51 [0.39 - 0.64] | 0.64 [0.49 - 0.87] | Overall p=.06  Subgroup analysis: active tumor vs LS p=.07  active tumors vs NHES p=.06 |
| Phase (radian) |  |  |  |  |  |
| -VLF | 0.93 [0.68 - 1.11] | 0.84 [0.50 - 1.01] | 0.75 [0.60 - 1.17] | 0.87 [0.68 - 1.21] | Overall p=.84 |
| -LF | 0.62 ]0.50 - 0.86] | 0.73 [0.49 - 0.95] | 0.74 [0.57 - 0.95] | 0.71 [0.40 - 0.89] | Overall p=.67 |
| -HF | 0.22 [0.04 - 0.34] | 0.30 [0.14 - 0.47] | 0.22 [0.13 - 0.44] | 0.11 [0.03 - 0.34] | Overall p=.26 |

Overall, Kruskal-Wallis test was used for comparisons over all groups; P=level of significance. CBFV, cerebral blood flow velocity in the middle cerebral artery; VLF, very low frequency; LF, low frequency; HF, high frequency. For end-tidal pCO2 and blood pressure values at the time of recordings, see Table 3. All data in median and interquartile range

Assessing dCA some confounders are to consider: first, EtCO2 which affects coherence, gain and phase; and secondly, coherence which is also a marker of the quality of the recordings. Even if coherence, gain or phase are not different between the groups, EtCO2 and coherence could influence the dCA parameter results within each stroke group. We, thus, controlled within each stroke group phase and gain for possible effects of EtCO2 and coherence by regression analysis models. To avoid redundance, we report only the significant result:

Effect of EtCO2 over the recording period: in the LS group, EtCO2 influenced gain in the VLF [AH: β=0.031 (95%CI 0.004 – 0.58), r^2^=0.08, F(2,43), p=0.03; UH: β=0.031 (95%CI 0.004 – 0.058), r^2^=0.09, F(2,42), p=0.04] only, gain and phase in all other frequency ranges remained unaffected. In the inactive tumor group, only phase in the LF range was affected by EtCO2 [AH: β= -0.011 (95%CI -0.001 – -0.021), r^2^= 0.211, F(2,18), p=0.05; UH: β=-0.016 (95%CI -0.004 - -0.028), r^2^=0.314, F(2,17), p=0.02], gain and phase in all other frequency ranges remained unaffected. In the NHES group and in the active tumor group EtCO2 did not affect gain and phase in any frequency range. EtCo2 was unrelated to coherence in all frequency ranges in both hemispheres. In the ANCOVA models the mean effects of ETCO2 on the phase/gain means were not different between the four stroke groups.

Effect of coherence: In the LS group, coherence was related only to HF gain in both hemispheres [AH: β=0.483 (95CI 0.124 – 0.745), r^2^=0.132, F(2,43), p=0.04; UH: β=0.552 (95%CI 0.062 – 0.972), r^2^=0.108, F(2,43), p=0.02]. In the NHES group, only HF gain in both hemispheres was related to coherence [AH: β=0.543 (95%CI 0.183 – 0.903), r^2^=0.165, F(2,37), p=0.006; UH: β=0.773 (95%CI 0.273 – 1.253), r^2^=0.237, F(2,37), p=0.003]. Coherence was related in the active tumor group only to HF gain in the unaffected hemisphere [β=1.096 (95%CI 0.197 – 1.996), r^2^=0.244, F(2,18), p=0.02], and in the inactive tumor group to HF gain in the affected hemisphere [β=0.622 (95%CI 0.164 – 1.082), r^2^=0.258, F(2,17), p=0.01]. In the ANCOVA models the mean effects of coherence on the phase/gain means were not different between the four stroke groups.

**Discussion**

The most constant differences between the four stroke groups were found in TFA gain. This is surprising as in ischemic stroke VLF or LF phase is usually the parameter who indicates group differences best [Panerai et al 2022; Nogueira et al 2022]. Because the amount of phase shift in ischemic stroke depends on infarct size (Reinhard et al, 2012; Nogueira et al; 2022) it seems plausible that our results are caused by the fact that infarct size was similar in our four stroke groups. The lack of distinct differences in the VLF and LF ranges in terms of gain and phase seems not to support our hypothesis that CBF(V) regulation in the tumor patients is different from the other stroke groups. However, LF and HF gain in the affected hemisphere were by trend higher in the active tumor group compared the LS group. It was also higher by trend in the HF range in the unaffected hemisphere. Compared to the NHES group, gain in the active tumor group was significantly or by trend higher in the HF range in both hemispheres. And compared to the inactive tumor group, the active tumor patients exhibited a higher gain in the HF range in both hemispheres.

In the high pass filter model of dCA, CBF(V) regulation takes place in the VLF and LF ranges. In the high frequencies BP changes (for example by heart beats) are passed on “unregulated” to CBF(V). As a result, the relationship over time between BP and CBFV is very constant indicated by a high coherence. However, this consistency over time accounts for only 25% or less of HF gain. As a consequence, 75% or more of HF gain variation is caused by other factors. Compared to the NHES patients, HF gain in the active tumor group was significantly (affected hemisphere) or short of significance (unaffected hemisphere) higher indicative for a higher generalized vascular tone or resistance. Compared to the LS group, gain in the active tumor group was also higher by trend in the LF and HF ranges, thus further supporting the assumption of a generalized vascular resistance involvement in the active tumor patients. An assumption that the HF gain result in the active tumor group is only an effect of arterial hypertension seems not plausible as the proportion of hypertensive patients in the inactive tumor group was equally high, but the HF gain in the inactive tumor group was significantly lower compared to the active tumor group. Potentially other mechanisms of a generalized vascular damage in active tumor patients could be the endothelial toxicity of chemotherapeutics which can among others cause vessel narrowing and could induce (speculatively) hereby a gain increase [Cadeddu et al, 2020; Hsu et al 2021; Campia,2020], or a tumor associated (paraneoplastic) inflammatory syndrome with or without disseminated intravascular hyper-coagulopathy [Dardiotis et al, 2019] with its endothelial-dependent pathophysiology. If confirmed in larger study populations, the strong trend that HF gain in the active tumor patients is higher than the one in the LS group, could signal a characteristic sign of cerebrovascular regulation in active tumor patients.

Our study is a retrospective one on routinely collected data with the result that the two tumor groups were small in number. We assume, that the trends in the HF gain would become significant with a larger study population. In a clearly designed prospective study an a-priori power calculation would have addressed the necessary size of the tumor groups more adequately. The state of the overall coagulation was not different in the four stroke groups; the inclusion of detailed coagulation analyses or the estimation of serologic parameters of endothelial functioning into our investigation would have been helpful to support or to reject our hypothesis that active tumor patients exhibit a generalized cerebrovascular dysfunction. Speculatively, autonomic nervous system disturbances, which could be part of the active tumor disease stage, can induce dCA changes, which are, however, found usually in the VLF [Hamner et al, 2010; Hamner et al, 2012; Lakatos et al, 2024] but not in the HF range. The CBF is regulated integrative by cerebral vessels with widely varying vessel diameters and varying functions over time [Schaeffer and Iadecola, 2021; Fan et al 2022]. We analyzed CBF velocity which is vessel diameter dependent, and assume that it is a close representative of CBF to allow for conclusions on CBF regulation. At present, our results are of limited clinical usefulness. If confirmed, they could be of help to characterize a defined subpopulation of stroke patients who, due to their poorer prognosis, need more attention in clinical research.

To summarize, we didn't find differences in cerebral regulation phase and gain in stroke patients with active tumor disease. However, we found the highest HF gain levels in the active tumor patient group, indicative for a generalized high vascular resistance, at least in this frequency range. Whether these changes are characteristic for this type of stroke source needs further research.

Conflict of Interests

No author declares a conflict of interest.

Author contributions

Lehel Barna Lakatos: writing, data collection

Manuel Bolognese: data analysis, important intellectual content.

Mareike Österreich: study concept, data collection

Martin Müller: writing, study concept

Grzegorz Marek Karwacki: analysis of magnetic resonance imaging.

References

Adams HP Jr, Bendixen BH, Kappelle LJ, Biller J, Love BB, Gordon DL, Marsh EE 3rd. Classification of subtype of acute is-chemic stroke. Definitions for use in a multicenter clinical trial. TOAST. Trial of Org 10172 in acute stroke treatment. Stroke 1993 Jan;24(1):35-41. doi: 10.1161/01.str.24.1.35; PMID: 7678184.

Ainslie PN, Celi L, McGrattan K, Peebles K, Ogoh S. Dynamic cerebral autoregulation and baroreflex sensitivity during modest and severe step changes in arterial PCO2. Brain Res. 2008 Sep 16;1230:115-24. doi: 10.1016/j.brainres.2008.07.048. Epub 2008 Jul 22. PMID: 18680730.

Bang OY, Chung JW, Lee MJ, Seo WK, Kim GM, Ahn MJ; OASIS-Cancer Study Investigators. Cancer-Related Stroke: An Emerging Subtype of Ischemic Stroke with Unique Pathomechanisms. J Stroke. 2020 Jan;22(1):1-10. doi: 10.5853/jos.2019.02278. Epub 2020 Jan 31. PMID: 32027788; PMCID: PMC7005348.

Cadeddu Dessalvi C, Deidda M, Giorgi M, Colonna P. Vascular Damage - Coronary Artery Disease. J Cardiovasc Echogr. 2020 Apr;30(Suppl 1):S11-S16. doi: 10.4103/jcecho.jcecho_3_19. Epub 2020 Apr 10. PMID: 32566461; PMCID: PMC7293870.

Campia U. Vascular effects of cancer treatments. Vasc Med. 2020 Jun;25(3):226-234. doi: 10.1177/1358863X20914978. PMID: 32539632.Dardiotis E, Aloizou AM, Markoula S, Siokas V, Tsarouhas K, Tzanakakis G, Libra M, Kyritsis AP, Brotis AG, Aschner M, Gozes I, Bogdanos DP, Spandidos DA, Mitsias PD, Tsatsakis A. Cancer-associated stroke: Pathophysiology, detection and management (Review). Int J Oncol. 2019 Mar;54(3):779-796. doi: 10.3892/ijo.2019.4669. Epub 2019 Jan 2. PMID: 30628661; PMCID: PMC6365034.Fan JL, Brassard P, Rickards CA, Nogueira RC, Nasr N, McBryde FD, Fisher JP, Tzeng YC. Integrative cerebral blood flow regulation in ischemic stroke. J Cereb Blood Flow Metab. 2022 Mar;42(3):387-403. doi: 10.1177/0271678X211032029. Epub 2021 Jul 14. PMID: 34259070; PMCID: PMC8985438.

Fazekas F, Barkhof F, Wahlund LO, Pantoni L, Erkinjuntti T, Scheltens P, Schmidt R. CT and MRI rating of white matter lesions. Cerebrovasc Dis. 2002; 13 Suppl 2: 31-6.

Hainsworth AH, Markus HS, Schneider JA. Cerebral Small Vessel Disease, Hypertension, and Vascular Contributions to Cognitive Impairment and Dementia. Hypertension. 2024 Jan;81(1):75-86. doi: 10.1161/HYPERTENSIONAHA.123.19943. Epub 2023 Nov 29. PMID: 38044814; PMCID: PMC10734789.

Hamner JW, Tan CO, Lee K, Cohen MA, Taylor JA. Sympathetic control of the cerebral vasculature in humans. Stroke. 2010 Jan;41(1):102-9. doi: 10.1161/STROKEAHA.109.557132. Epub 2009 Dec 10. PMID: 20007920; PMCID: PMC2814242.

Hamner JW, Tan CO, Tzeng YC, Taylor JA. Cholinergic control of the cerebral vasculature in humans. J Physiol. 2012 Dec 15;590(24):6343-52. doi: 10.1113/jphysiol.2012.245100. Epub 2012 Oct 15. PMID: 23070700; PMCID: PMC3533196

Hsu PY, Mammadova A, Benkirane-Jessel N, Désaubry L, Nebigil CG. Updates on Anticancer Therapy-Mediated Vascular Toxicity and New Horizons in Therapeutic Strategies. Front Cardiovasc Med. 2021 Jul 27;8:694711. doi: 10.3389/fcvm.2021.694711. PMID: 34386529; PMCID: PMC8353082.

Lakatos L, Bolognese M, Müller M, Österreich M, von Hessling A. Automated supra- and in-fratentorial brain infarct volume estimation on diffusion weighted imaging using the RAPID software. Front Neurol. 2022 8; 13:907151. doi: 10.3389/fneur.2022.907151. PMID: 35873774; PMCID: PMC9304979.

Lakatos LB, Shin DC, Müller M, Österreich M, Marmarelis V, Bolognese M. Impaired dynamic cerebral autoregulation measured in the middle cerebral artery in patients with vertebrobasilar ischemia is associated with autonomic failure. J Stroke Cerebrovasc Dis. 2024 Jan;33(1):107454. doi: 10.1016/j.jstrokecerebrovasdis.2023.107454. Epub 2023 Nov 4. PMID: 37931481.

Lipsitz LA, Mukai S, Hamner J, Gagnon M, Babikian V. Dynamic regulation of middle cerebral artery blood flow velocity in aging and hypertension. Stroke. 2000 Aug;31(8):1897-903. doi: 10.1161/01.str.31.8.1897. PMID: 10926954.

Lyden P, Brott T, Tilley B, Welch KM, Mascha EJ, Levine S, Haley EC, Grotta J, Marler J. Improved reliability of the NIH Stroke Scale using video training. NINDS TPA Stroke Study Group. Stroke. 1994; 25: 2220-6.

Markus HS, de Leeuw FE. Cerebral small vessel disease: Recent advances and future directions. Int J Stroke. 2023 Jan;18(1):4-14. doi: 10.1177/17474930221144911. PMID: 36575578; PMCID: PMC9806465.

Müller MW, Osterreich M. A comparison of dynamic cerebral autoregulation across changes in cerebral blood flow velocity for 200 s. Front Physiol. 2014 Aug 26;5:327. doi: 10.3389/fphys.2014.00327. PMID: 25206340; PMCID: PMC4144203.

Müller M, Österreich M, von Hessling A, Smith RS. Incomplete recovery of cerebral blood flow dynamics in sufficiently treated high blood pressure. J Hypertens. 2019 Feb;37(2):372-379. doi: 10.1097/HJH.0000000000001854. PMID: 29995701; PMCID: PMC6365247.

Navi BB, Kasner SE, Elkind MSV, Cushman M, Bang OY, DeAngelis LM. Cancer and Embolic Stroke of Undetermined Source. Stroke. 2021 Mar;52(3):1121-1130. doi: 10.1161/STROKEAHA.120.032002. Epub 2021 Jan 28. PMID: 33504187; PMCID: PMC7902455.

Nogueira RC, Aries M, Minhas JS, H Petersen N, Xiong L, Kainerstorfer JM, Castro P. Review of studies on dynamic cerebral autoregulation in the acute phase of stroke and the relationship with clinical outcome. J Cereb Blood Flow Metab. 2022 Mar;42(3):430-453. doi: 10.1177/0271678X211045222. Epub 2021 Sep 13. PMID: 34515547; PMCID: PMC8985432

Panerai RB, Deverson ST, Mahony P, Hayes P, Evans DH. Effects of CO2 on dynamic cerebral autoregulation measurement. Physiol Meas. 1999 Aug;20(3):265-75. doi: 10.1088/0967-3334/20/3/304. PMID: 10475580.

Panerai RB, Brassard P, Burma JS, Castro P, Claassen JA, van Lieshout JJ, Liu J, Lucas SJ, Minhas JS, Mitsis GD, Nogueira RC, Ogoh S, Payne SJ, Rickards CA, Robertson AD, Rodrigues GD, Smirl JD, Simpson DM; Cerebrovascular Research Network (CARNet). Transfer function analysis of dynamic cerebral autoregulation: A CARNet white paper 2022 update. J Cereb Blood Flow Metab. 2023 Jan;43(1):3-25. doi: 10.1177/0271678X221119760. Epub 2022 Aug 12. PMID: 35962478; PMCID: PMC9875346.

.

Pires PW, Dams Ramos CM, Matin N, Dorrance AM. The effects of hypertension on the cerebral circulation. Am J Physiol Heart Circ Physiol. 2013 Jun 15;304(12):H1598-614. doi: 10.1152/ajpheart.00490.2012. Epub 2013 Apr 12. PMID: 23585139; PMCID: PMC4280158.

Reinhard M, Rutsch S, Lambeck J, Wihler C, Czosnyka M, Weiller C, Hetzel A. Dynamic cerebral autoregulation associates with infarct size and outcome after ischemic stroke. Acta Neurol Scand. 2012 Mar;125(3):156-62. doi: 10.1111/j.1600-0404.2011.01515.x. Epub 2011 Apr 6. PMID: 21470192.

Schaeffer S, Iadecola C. Revisiting the neurovascular unit. Nat Neurosci. 2021 Sep;24(9):1198-1209. doi: 10.1038/s41593-021-00904-7. Epub 2021 Aug 5. PMID: 34354283; PMCID: PMC9462551.

Serrador JM, Sorond FA, Vyas M, Gagnon M, Iloputaife ID, Lipsitz LA. Cerebral pressure-flow relations in hypertensive elderly humans: transfer gain in different frequency domains. J Appl Physiol (1985). 2005 Jan;98(1):151-9. doi: 10.1152/japplphysiol.00471.2004. Epub 2004 Sep 10. PMID: 15361517.

Van Swieten JC, Koudstaal PJ, Visser MC, Schouten HJ, van Gijn J. Interobserver agreement for the assessment of handicap in stroke patients. Stroke. 1988; 19: 604-7.

Zhang R, Witkowski S, Fu Q, Claassen JA, Levine BD. Cerebral hemodynamics after short- and long-term reduction in blood pressure in mild and moderate hypertension. Hypertension. 2007 May;49(5):1149-55. doi: 10.1161/HYPERTENSIONAHA.106.084939. Epub 2007 Mar 12. PMID: 17353511.

.
